# The Role of Dietary Magnesium in Cardiovascular Disease

**DOI:** 10.3390/nu16234223

**Published:** 2024-12-06

**Authors:** Forrest H. Nielsen

**Affiliations:** Center for Magnesium Education and Research, Pahoa, HI 96778, USA; frostynielsen@yahoo.com; Tel.: +1-701-775-2798

**Keywords:** magnesium deficiency, cardiovascular disease, inflammatory stress, magnesium requirements

## Abstract

In the past 20 years, a large number of epidemiological studies, randomized controlled trials, and meta-analyses have found an inverse relationship between magnesium intake or serum magnesium and cardiovascular disease, indicating that low magnesium status is associated with hypertension, coronary artery calcification, stroke, ischemic heart disease, atrial fibrillation, heart failure, and cardiac mortality. Controlled metabolic unit human depletion–repletion experiments found that a mild or moderate magnesium deficiency can cause physiological and metabolic changes that respond to magnesium supplementation, which indicates that these types of deficiencies or chronic latent magnesium deficiency are contributing factors to the occurrence and severity of cardiovascular disease. Mechanisms through which a mild or moderate magnesium deficiency can contribute to this risk include inflammatory stress, oxidative stress, dyslipidemia and deranged lipid metabolism, endothelial dysfunction, and dysregulation of cellular ion channels, transporters, and signaling. Based on USA official DRIs or on suggested modified DRIs based on body weight, a large number of individuals routinely consume less magnesium than the EAR. This especially occurs in populations that do not consume recommended amounts of whole grains, pulses, and green vegetables. Thus, inadequate magnesium status contributing to cardiovascular disease is widespread, making magnesium a nutrient of public health concern.

## 1. Introduction

The realization that inadequate magnesium status not caused by predisposing conditions causing impaired absorption or increased loss can contribute to cardiovascular disease is a rather recent phenomenon. This realization was caused by the dogma that the body has mechanisms that, when magnesium intakes are low, increase the percentage of ingested magnesium absorbed, reduce magnesium loss in urine, and use magnesium reserves in the bone to maintain levels needed for its numerous biochemical functions. This dogma resulted in numerous opinions about the limited importance of nutritional magnesium for the general public. In 2004, an expert consultation for the Food and Agriculture and World Health Organization concluded that evidence was lacking for nutritional magnesium deficiency with dietary intakes less than the current Dietary Reference Intakes (DRIs) of the United States, Canada, and Europe [1]. In 2010, The Dietary Guidelines for Americans’ position on magnesium was that it was not a major nutrient of concern for health and well-being beyond those individuals taking medications or having disorders that inhibit magnesium absorption or increase its excretion [2]. As late as 2014, The Harvard Medical School’s HEALTHbeat advisory [3] stated that “Magnesium deficiency is very rare”. It is only in the last 10 years that serious attention has been given to inadequate magnesium status having an impact on the incidence of cardiovascular disease in the general population.

## 2. Initial Studies Implicating Magnesium Deficiency in Cardiovascular Disease

Magnesium was generally accepted as essential for animals and humans before direct proof of its essentiality was provided by the induction of magnesium deficiency in animals and humans. In 1932, magnesium deficiency was induced in rats, with one of the symptoms being cardiac arrhythmia [4]. Although some signs and symptoms occurring in predisposing diseases affecting the intake, absorption, or excretion of magnesium were ascribed to magnesium deficiency, the first experimentally verifiable human magnesium deficiency was not reported until 1969 [5]. This experiment used a diet that was essentially made of purified ingredients that had to be provided by a nasoesophageal tube because of carcinoma treatment in the oral cavity. It was concluded that all symptoms occurred secondary to complex electrolyte changes, mainly alterations in calcium and potassium. Among those changes were alterations in electrocardiograms. These first studies did not raise any alarm about magnesium deficiency being a factor in the incidence or severity of cardiovascular disease.

In 1974, a review of animal findings and some limited human findings, in addition to the finding that magnesium in hard water was associated with reduced ischemic heart disease [6], indicated that magnesium affects cardiovascular health not only at the cellular level but also through an effect on lipid metabolism and coagulation/fibrinolytic mechanisms. However, this review did not stimulate the concept that magnesium deficiency in the general population might be a contributor to cardiovascular disease. Seelig, in 1981 [7], stated that most physicians did not accept the likelihood of marginal magnesium deficiency being a possible contributory factor to the early origins or later manifestations of cardiovascular disease. Reviews in 1976 [8] and 1981 [9] indicated that magnesium deficiency occurred mainly in individuals with disease states affecting magnesium absorption and excretion. These reviews list a large number of these conditions that cause magnesium deficiency, and the manifestation of this deficiency in cardiovascular health was limited to abnormal electrocardiograms, premature ventricular beats, ventricular tachycardia, and ventricular fibrillation. They also reported that pharmacological amounts of magnesium were used to counteract cardiac arrhythmias.

## 3. Fundamental Semination Promulgating Magnesium Need for Cardiovascular Health

In 1988, Elin introduced the term chronic latent magnesium deficiency as an individual with a reduction in total body magnesium but with a serum total magnesium content within an accepted reference interval [10]. The term chronic indicated that this status could take a long period of time to occur. Chronic latent magnesium deficiency is prevalent in humans [11], which indicates that many individuals could have an inadequate magnesium status that could contribute to the occurrence of cardiovascular disease. In 1993, a second experimental magnesium deficiency was induced in humans by feeding a liquid diet that contained 0.5 mmol (121.5 mg)/day magnesium [12], an amount that is near what some individuals might routinely consume. This deficiency increased angiotensin-induced plasma aldosterone and thromboxane synthesis, which suggested an effect on vascular disease.

In the late 1990s and early 2000s, a series of metabolic unit experiments were conducted to test the hypothesis that naturally occurring inadequate intakes of magnesium resulted in negative magnesium balance and inadequate magnesium status such that metabolic and physiologic changes occurred that responded to magnesium supplementation. In addition to getting results to support the hypothesis, many of the changes found in healthy postmenopausal women were in variables associated with cardiovascular function. When consuming diets comprised of conventional foods providing between 100 and 118 mg/2000 kcal, these changes included adversely affecting cardiovascular function during submaximal work [13], an increase in both supraventricular and supraventricular plus ventricular beats [14], decreased urinary calcium excretion and increased calcium balance and thus increased calcium retention [15,16], decreased urinary excretion of phosphorus and potassium [17], altered oxidative metabolism, and induced heart rhythm changes, including atrial fibrillation [17]. Also, during this time, epidemiologic and clinical evidence that chronic latent magnesium deficiency was associated with hypertension was receiving attention [18,19].

After 2006, an increasing number of epidemiological studies, randomized controlled trials, and meta-analyses provided much evidence that magnesium intake or serum magnesium was inversely associated with cardiovascular disease. Many of the first waves of these reports showing that magnesium status was inversely related to hypertension, coronary artery calcification, stroke, ischemic heart disease, atrial fibrillation, heart failure, and cardiac mortality have been described in several reviews [20,21,22,23,24].

Table 1 lists many of these types of studies reported since 2018. In addition to healthy populations, a low magnesium status has been associated with cardiovascular disease in patients with health problems such as diabetes and chronic kidney disease. For example, systemic reviews and meta-analyses have found an inverse relation between magnesium intake and stroke [25] and between serum magnesium and all-cause cardiovascular mortality in chronic kidney disease and end-stage renal disease patients [26]. An umbrella meta-analysis of randomized controlled trials found that magnesium supplementation decreased both systolic and diastolic blood pressure in an analysis that included 8610 subjects [27]. The decrease was particularly effective at doses of ≥400 mg/day for ≥12 weeks. A prospective study with a total of 149,929 participants found that a sufficient dietary intake was associated with lower risks of atherosclerotic cardiovascular disease and mortality in those with type 2 diabetes [28].

## 4. Mechanisms Through Which Chronic Latent Magnesium Deficiency Is Linked to Cardiovascular Disorders

Animal, cellular, and human studies have provided several mechanisms through which magnesium deficiency can contribute to the occurrence of cardiovascular disease. These mechanisms are based on magnesium having a vital role in metabolic pathways as a cofactor for over 600 enzymatic reactions that include those involved in lipid metabolism and cellular messenger systems. Magnesium also regulates cellular ion channels, transporters, and signaling that govern calcium, potassium, and sodium movement in and out of the cell. As a result, magnesium is a controlling factor in smooth muscle contraction, cardiac excitability, vasomotor tone, and blood pressure. Numerous reports have indicated individuals with chronic latent magnesium deficiency have changes in the mechanisms that are important for cardiovascular health.

## 5. Inflammatory Stress

Chronic inflammatory stress is characterized by abnormal cytokine production, increased acute-phase reactants, and activation of a network of inflammatory signaling pathways [45]. A recent review [46] references in vitro and animal experiments showing that magnesium deprivation increased the inflammatory cytokines TNF-α, IL-6, IL-1β, and plasminogen activator inhibitor-1, and the acute phase proteins fibrinogen, α2-macroglobulin, and complement. In addition, these studies found that magnesium deprivation activated phagocytic cells, opened calcium cellular channels, activated the NMDA receptor and NF-κB-signaling, caused dysbiosis, and increased cellular senescence, which could lead to inflammatory stress.

Human studies indicating that inadequate magnesium status is associated with inflammatory stress are limited to mostly those showing an increase in C-reactive protein (CRP), which is an accepted marker of ongoing inflammation or chronic inflammatory stress. The American Heart Association has stated that a plasma concentration of 3.0 mg/L CRP is the threshold that indicates chronic inflammatory stress is associated with increased cardiovascular disease risk [47]. A report that lists most of the studies showing the relationship between magnesium and CRP included a dataset from seven cross-sectional studies with a total of 32,918 individuals and five intervention studies [48]. A meta-analysis of the cross-sectional data set found an inverse relationship between magnesium intake and serum CRP concentration. Three of the studies provided data that showed the pooled odds ratio (95% Confidence Interval) of having CRP ≥ 3.0 mg/L was 1.49 when comparing the lowest to the highest magnesium intake. In the intervention studies, magnesium supplementation alleviated elevated CRP; these studies involved participants with deficient serum magnesium concentrations that were increased by magnesium supplementation. The two studies that did not show a significant effect of magnesium supplementation on serum CRP concentrations involved participants without elevated serum CRP concentrations, and magnesium supplementation did not increase serum magnesium. A systematic review and meta-analysis of randomized controlled trials revealed the same effect of magnesium supplementation on plasma CRP [49]. In 11 studies, magnesium supplementation did not significantly affect plasma CRP concentrations. However, when the analysis was stratified to compare subgroups with CRP values ≤ 3 with those >3, magnesium treatment significantly reduced CRP in the latter group but not in the former group. Actually, not finding an effect of magnesium supplementation on serum plasma CRP concentration when they are less than 3 mg/L supports the concept that chronic latent magnesium deficiency may lead to chronic inflammatory stress because it is likely that chronic inflammatory stress was not present when magnesium may have been adequate. A more recent meta-analysis of randomized controlled trials also indicated that magnesium supplementation in likely magnesium-deficient individuals decreased serum CRP and nitric oxide levels [50]. The inflammatory stress related to chronic latent magnesium deficiency can adversely affect heart health in several different ways, including oxidative damage, deranged lipid metabolism, and altered endothelial function.

## 6. Oxidative Stress

Oxidative stress is an imbalance between oxidants (reactive oxygen and nitrogen species) and antioxidants. Excessive reactive oxygen species cause increased peroxidation of molecules and tissues, which generates tissue necrosis, membrane oxidation, cellular apoptosis, and atherosclerotic lesions. In vitro and animal studies have shown possible mechanisms through which magnesium deficiency can induce oxidative stress. Mitochondria are the primary source of reactive oxygen species. As described in a recent review, intracellular magnesium deficiency inhibits magnesium transport into mitochondria, resulting in mitochondrial magnesium deficiency that decreases electron transport chain activity and alters coupled respiration, which increases the production of reactive oxygen species [51]. In addition, antioxidants such as superoxide dismutase and mitochondrial glutathione are suppressed. The decreased mitochondrial activity also downregulates ATP synthase, which results in decreased ATP concentration, which causes an increase in nicotinamide adenine dinucleotide phosphate (NADPH) oxidase activity [51]. NADPH oxidase stimulates macrophages and neutrophils to generate reactive oxygen species. This review also described mechanisms through which magnesium deficiency increases mitochondrial and intracellular calcium [51]. An increase in intracellular calcium prompts the release of inflammatory cytokines and activates NADPH oxidase and nitric oxide synthase, which promotes the generation of reactive oxygen species.

There are a few human studies indicating that chronic latent magnesium deficiency can result in oxidative stress. Metabolic unit experiments found that post-menopausal women fed Western diets providing 100 mg/2000 kcal for 78–81 days had erythrocyte superoxide dismutase concentrations that decreased during the deprivation period and increased during the 58–81 days when the diets were supplemented with magnesium at either 399 or 300 mg/2000 kcal [17,52]. The difference in concentration found at the end of each dietary period was significant. A meta-analysis of 194 participants in three randomized control trials found that magnesium supplementation significantly increased serum nitric oxide, an anti-inflammatory mediator [50].

## 7. Dyslipidemia and Deranged Lipid Metabolism

Animal studies indicate that magnesium deficiency-induced dyslipidemia contributes to atherosclerotic vascular disease [19]. The dyslipidemia included serum and plasma increased phospholipid and triglyceride concentrations, variable cholesterol concentrations, and modifications in lipoproteins [19]. Magnesium deficiency also increased triglyceride-rich lipoproteins that were associated with an increase in plasma apo B and a decrease in apo A1 and apo E [53]. In the heart, magnesium deficiency increases the oxidation of lipids and lipoproteins [19]. Modified lipid concentrations and oxidation in plasma and heart are considered contributors to the development of atherosclerotic lesions.

Two metabolic unit magnesium depletion–repletion experiments in postmenopausal women who were placed shortly after arrival on a magnesium-deficient diet have indicated that a chronic latent magnesium deficiency can induce lipid changes, some of which could contribute to the occurrence of cardiovascular disease. In one of these experiments, while feeding on a Western diet containing about 100 mg/2000 kcal magnesium for 81 days, serum triglycerides and total and LDL-cholesterol concentrations decreased and then increased during the 58-day period when dietary magnesium was increased to 300 mg/2000 kcal [17]. The difference in concentration found at the end of each dietary period was significant. A similar result was found when the diet containing 100 mg/2000 kcal was consumed for 84 days, or 42 days followed by consumption of diets containing about 400 mg/2000 kcal. There was no effect of HDL-cholesterol in either experiment. In one experiment, magnesium supplementation after magnesium deprivation increased serum Apo A1 and Apo B concentrations [15]. These findings contrast sharply with those obtained in rats [53]. Severe acute magnesium deficiency increased serum triglycerides and Apo B and decreased HDL-cholesterol and Apo A1 concentrations in rats. The contrasting results suggest that the severity of magnesium deficiency and the length of time of magnesium deprivation affect triglyceride, cholesterol, and lipoprotein changes. These changes might reflect the extent of the effects magnesium deficiency has on other roles these metabolites play throughout the body beyond those that can affect atherosclerotic vascular disease.

## 8. Endothelial Dysfunction

Studies of a model of familial hypomagnesemia in mice and human umbilical vascular endothelial cells (HUVECs) provide evidence that chronic latent magnesium deficiency can cause a disorder of endothelial function, which is a key player in the initial steps in the genesis of atherosclerosis, coronary heart disease, and hypertension. Binding of mononuclear leukocytes and T lymphocytes to the intima is an early event in atherosclerosis [54]. The bound leukocytes penetrate and become resident in the vascular wall and the monocytes differentiate into macrophages that scavenge modified lipoproteins and lipids to become foam cells. These cells are the precursors of the fatty streak that can evolve into a more fibrous and complicated plaque that can cause clinical disease [54]. Studies with HUVECs have found that dysfunctional endothelial cells, which line the inner walls of blood vessels, are critical players in this process and are highly responsive to magnesium deficiency [55,56]. Magnesium supplementation has been shown to significantly improve endothelial function in patients with ischemic heart disease [57]. One endothelial cell response to magnesium deficiency that indicates dysfunction is an effect on inducible NO synthase (iNOS) [56]. Nitric oxide controls endothelial cell permeability and adhesiveness, but excessive amounts enhance reactive oxygen formation. Vascular NO production by iNOS in endothelial cells is increased by magnesium deficiency [56]. Only under low magnesium conditions can pathways lead to the accumulation of reactive oxygen species in endothelial cells [56]. Nitric oxide produced by endothelial nitric oxide synthase (eNOS) relaxes smooth muscle, controls endothelial cell permeability and adhesiveness, has antioxidant properties, and is enhanced under high magnesium conditions. As described above, a meta-analysis of three controlled randomized trials found that magnesium supplementation increased serum nitric oxide [50]. This study, plus those showing magnesium supplementation improved endothelial function in ischemic heart disease [57], provides further evidence that chronic latent magnesium deficiency is contributing to the incidence and severity of cardiovascular disease.

## 9. Dysregulation of Cellular Ion Channels, Transporters, and Signaling

Another mechanism through which chronic latent magnesium deficiency can affect inflammatory and oxidative stress, leading to cardiovascular disease, is through affecting electrolyte metabolism that results in dysregulation of cellular ion channels, transporters, and signaling. Animal studies have indicated that magnesium deficiency primes phagocytic cells (e.g., granulocytes, macrophages) to release proinflammatory cytokines [58]. A primary factor for this priming is an increase in intracellular calcium. Magnesium has been called nature’s physiological calcium channel blocker [19]. In magnesium deficiency, cellular calcium increases through an influx from extracellular sources via slow calcium transport channels and release from intracellular sources such as the sarcoplasmic reticulum. Increased intracellular calcium induces the release of inflammatory cytokines such as IL-1, IL-6, and TNF-α; leukotrienes neuropeptides such as substance P; and prostaglandins such as prostaglandin E_2_.

In addition to affecting inflammatory stress, the dysregulation of electrolyte metabolism can affect cardiovascular disorders, hypertension, and cardiac arrhythmia. Increased intracellular calcium caused by magnesium deficiency results in increased vascular smooth muscle and myocyte constriction and vascular tone. Altered vascular tone leads to hypertension [59], and altered coronary vascular tone has a role in myocardial infarction [19].

Hypokalemia is common in patients with severe hypomagnesemia. However, the promotion of potassium depletion from the body by magnesium deprivation can result in reduced intracellular and total body potassium even when plasma or serum potassium is not appreciably reduced [59]. Intracellular potassium depletion is apparently involved in the dysrhythmias that can be present in patients with magnesium deficiency. These arrhythmias include atrial fibrillation, ventricular premature contractions, and junctional arrhythmias [19].

Some evidence exists that indicates that a chronic latent magnesium deficiency can alter electrolyte metabolism such that it can contribute to cardiovascular disease. Under a controlled metabolic unit environment, women were fed a diet composed of ordinary Western foods that provided magnesium at 118 and 318 mg/day [15]. Seven women consumed the magnesium-deficient diet for consecutive 42-day periods (84 days), and six women consumed the magnesium-deficient diet for 42 days that alternated with 42 days of consuming a magnesium-adequate diet. Consuming the low magnesium diet decreased urinary calcium excretion, increased calcium balance, and decreased urinary potassium excretion [15]. Serum potassium was not significantly affected by magnesium deprivation. After 72 days of consuming the low-magnesium diet, one woman exhibited heart ventricular ectopy that disappeared after consuming the magnesium-adequate diet. In another metabolic unit study, women were fed diets with mean magnesium intakes of 107 and 327 mg/day for 72 days in a crossover design [16]. In this study, magnesium deprivation increased calcium balance and decreased the urinary excretion of calcium and potassium. In a third controlled environment metabolic unit study, postmenopausal women were fed a diet composed of Western foods that provided magnesium at 101 mg/2000 kcal for 78 days, and then the diet was supplemented with 200 mg/2000 kcal of magnesium [17]. In five of fourteen women, heart rhythm changes occurred that included atrial flutter, atrial fibrillation, ventricular premature beats, and second-degree block during magnesium deprivation. Magnesium deprivation decreased urinary potassium but did not affect serum potassium. In another report involving two controlled metabolic unit studies in which postmenopausal women were fed less than one-half of or more than the recommended dietary allowance (RDA) of magnesium (320 mg/day) in a double-blind crossover design, magnesium deprivation significantly increased both supraventricular and supraventricular plus ventricular beats [14]. Hypomagnesemia, hypocalcemia, and hypokalemia were not found.

The decreased urinary excretion of calcium and potassium contrasts with the increased urinary excretion found with severe magnesium deficiency in humans, which is indicated by hypocalcemia and hypokalemia. In magnesium deprivation severe enough to cause hypomagnesemia, hypokalemia and hypocalcemia are frequently found [60]. When magnesium deficiency is severe enough to cause hypomagnesemia, it has profound effects on calcium by affecting parathyroid use and on potassium by affecting Na^+^-K^+^-ATPase pump activity. Changes were not found with parathyroid metabolism, nor were hypocalcemia and hypokalemia in the metabolic unit studies. This suggests that the signs of electrolyte metabolism change as the magnesium deficiency progresses from chronic latent magnesium or mild to moderate deficiency to magnesium deficiency severe enough to cause overt signs of hypomagnesemia. For example, mild or moderate magnesium deficiency might cause increased intracellular calcium by retaining calcium through reduced calcium excretion while assuring that plasma concentrations were not affected. An animal study supports the finding that increased retention and decreased excretion occur in mild or moderate magnesium deficiency. Calcium retention and balance and urinary excretion decreased in old rats fed a moderately magnesium-deficient diet for four weeks [61]. In the case of potassium, decreased urinary excretion of potassium might reflect the body’s attempt to retain it for critical biochemical functions that were suppressed by intracellular magnesium deficiency. Thus, the controlled environment metabolic unit findings support the posit that chronic latent magnesium deficiency or mild to moderate magnesium deficiency can cause changes to electrolyte metabolism that contribute to the incidence of cardiovascular disease.

## 10. Magnesium Intake for the Prevention of Cardiovascular Disease

Because magnesium has so many critical functions, the body has mechanisms to ensure it is available to perform them [24]. During low intakes of magnesium, the percentage absorbed from the diet is increased, the amount excreted in the urine is decreased, and body reserves, especially in bones, are accessed. When dietary magnesium provides more than sufficient magnesium for body functions, the opposite occurs. The response of the body to maintain magnesium homeostasis has made it difficult to establish dietary requirements for magnesium. In 1997, the United States set the Estimated Average Requirement (EAR) and Recommended Dietary Allowance (RDA) for adult women at 255–265 mg/day and 310–320 mg/day, respectively [62]. The EAR and RDA for adult men were set at 330–350 mg/day and 410–420 mg/day, respectively. These Dietary Reference Intakes (DRIs) were based on highly variable balance data from 16 men and 18 women on self-selected diets with relatively low magnesium content during the balance determinations. Since these EARs and RDAs were established, balance data from a larger number of subjects under controlled dietary and environmental conditions of a metabolic unit have given better evidence-based values for magnesium EARs and RDAs. These studies included 93 men and 150 women aged 19–77 years and weighing 46 to 136 kg (average weight 76 kg) who were fed dietary magnesium ranging from 84 to 598 mg/day [63]. The subjects were not obese, subjected to any major stress, and were neither taking drugs nor had underlying health conditions that could affect magnesium requirements. The metabolic unit provided strict control of food consumption, weight, exercise, and fecal and urine collection.

Data obtained from the metabolic unit studies found that neutral magnesium balance occurred at an intake of 165 mg/day with a 95% prediction interval of 113 and 237 mg/day. Using the upper value of 237 mg/day and considering that 98% is the upper interval level used for setting the RDAs would result in an RDA of 245 mg/day for both adult men and women. Considering surface, phlebotomy, menstrual, and seminal losses that were not measured would likely increase the RDA to 250 mg/day [64]. Using the value of 165 mg/day and the same manipulations as conducted for the RDA would yield an ERA of 175 mg/day. Balance data from German men and women support these EAR and RDA for magnesium [65]. The German data indicated normative magnesium requirements were less than 200 mg/day for women and 250 mg/day for men.

However, it should be realized that ERA and RDA could be much higher than that indicated by the more controlled balance studies for many individuals in the general population. The metabolic unit balance data found a neutral magnesium balance per kg of body weight of 2.36 mg/day, with a 95% prediction interval of 1.58 and 3.38 mg/day [63]. These data indicate that individuals weighing more than 76 kg (average weight of the subjects) should have EARs and RDAs higher than 175 and 250 mg/day, respectively. For example, the suggested RDA for a 100 kg (220 pounds) adult would be 335 mg/day, and the EAR would be 215 mg/day. The 2020–2025 dietary guidelines (DGAs) [66] stated that about 74% of adults are overweight or obese, with over 40% of adults over the age of 40 being obese, which would increase the EARs and RDAs for this large number of individuals. This increase is supported by reports that a low magnesium status occurs more often in obese than non-obese individuals [67,68]. In addition to weight, magnesium requirements could be increased by other factors such as psychological stress, high calcium intake, and high fiber/phytate intake [24,69]. Other inducers of inflammatory and oxidative stress could also affect the DRIs for adults. Peer-reviewed publications through 2010 have associated 45 dietary factors with serum concentrations of six inflammatory markers [70]. Many of these dietary factors could either alleviate or enhance the requirement for magnesium needed to reduce inflammatory and oxidative stress caused by mild or moderate magnesium deficiency or chronic latent magnesium deficiency and thus the risk of cardiovascular disease [71]. Further evidence that other factors can increase the requirement for magnesium is the latest suggested status indicator, the magnesium depletion score. This indicator is based on the use of diuretics, proton pump inhibitors, alcohol consumption, and kidney function [38]. The magnesium score has been associated with various disorders associated with inflammatory and oxidative stress, including cardiovascular disease [29,40,42,44]. Because so many factors can affect the magnesium requirement for an individual, setting one value each for RDA and EAR for magnesium to be used by an individual to reduce the risk of cardiovascular disease is difficult. However, if one value has to be established, consideration should be made to make it much higher than the EAR and RDA found for healthy individuals with minimal psychological and nutritional stressors on the requirement. Consideration could be given to stating the need for magnesium as has been done for potassium, where a requirement is given, but a much higher amount is recommended to prevent cardiovascular disease. Because high magnesium intake from food has not been found to cause adverse effects, the RDAs and EARs established for magnesium in 1997 might be close to intakes people should strive to achieve to effectively reduce the risk of cardiovascular disease.

## 11. Occurrence of Magnesium Deficiency That Could Affect the Risk of Cardiovascular Disease

Based on the current DRIs, surveys indicate a large number of individuals routinely have deficient intakes of magnesium. A comparison between the DRIs and magnesium intakes using NHANES 2011–2014 data found that the total intake of magnesium (food, beverages, supplements) was below the EAR for 45.2% for all adults [72]. Just considering the usual magnesium intake from food alone by adults over the age of 19 years, 52.0% of men and 50.7% of women had intakes that were below the EAR [72]. These values are similar to those found in the NHANES 2005–2006 survey [73]. Using these data, which break down the intakes more, even if the EARs and RDAs found for 76 kg adults under minimal additional nutritional, health, and psychological stress, the survey indicates that almost 50% of adult females and 25% of adult males would have intakes less than the suggested RDA of 250 mg/day. Because many individuals weigh more than 76 kg, it is likely that more than 25% of adults have usual intakes that could result in chronic latent magnesium deficiency or mild to moderate magnesium deficiency that can contribute to the risk of cardiovascular disease. It is not surprising that a large number of adults commonly consume inadequate amounts of magnesium. The best food sources for magnesium are green leafy vegetables, seeds, whole grains, pulses, and nuts [24,74]. The 2020–2025 DGA reported that the average adult consumes much less than the recommended daily intake of whole grains, green vegetables, and pulses [66].

## 12. Conclusions

Epidemiological studies, randomized controlled trials, meta-analyses, and controlled metabolic unit depletion–repletion experiments, with support from animal and in vitro findings, provide overwhelming evidence that mild to moderate magnesium deficiency and chronic latent magnesium deficiency are major contributors to the occurrence or the severity of cardiovascular disease. Because a large number of individuals do not routinely consume foods that provide the requirement to prevent this deficiency, magnesium is a nutrient of public health concern.

## Figures and Tables

**Table 1:** Recent reports showing that magnesium status is inversely correlated with cardiovascular disease.

Reference	Study Type	Number of Subjects	Magnesium Indicator	Lowest Indicator Level	Highest Indicator Level
Hypertension					
Tan et al., 2024 [29]	Cross-sectional	9708	Magnesium Depletion Score	≤2	≥3
Han et al., 2024 [30]	Cross-sectional	24,171	Dietary Magnesium	Quintile	Quintile
Stroke					
Zhao et al., 2019 [31]	Systematic Review and Meta-Analysis	692,998	Dietary Magnesium	<180 mg/day	≥180 mg/day
Sun et al., 2023 [32]	Cross-Sectional	29,653	Dietary Magnesium	Quartile < 188.5 mg/day	Quartile ≥ 362 mg/day
Dong et al., 2024 [33]	Mendelian Randomization	≥150,765	Dietary Intake	ND	ND
Coronary and Ischemic Heart Disease					
Gant et al., 2018 [34]	Cross-Sectional	450	Plasma Magnesium Urinary Magnesium Dietary Magnesium	Quartile 0.67 mmol/L 1.81 mmol/24 h 254 mg/day	Quartile 0.88 mmol/L 6.64 mmol/24 h 361 mg/day
Zhao et al., 2019 [35]	Meta-Analysis	554,581	Serum Magnesium Dietary Magnesium		
Rooney et al., 2020 [36]	Prospective and Meta-Analysis	14,446	Serum Magnesium	Quintile 1.45 mEq/L	Quintile 1.80 mEq/L
Yang et al., 2024 [37]	Meta-Analysis	2980	Dietary Magnesium	≤186 mg/day	≥357 mg/day
Atrial Fibrillation					
Wu et al., 2024 [38]	Observational	15,792	Serum Magnesium	≤1.49 mg/dL	≥1.90 mg/dL
Heart Failure					
Wannamethee et al., 2018 [39]	Prospective	3523	Serum Magnesium	Quintile < 0.75 mmol/L	Quintile ≥ 0.87 mmol/L
Zhao et al., 2024 [40]	Cross-Sectional	19,227	Magnesium Depletion Score	≥2	≥3
Cardiac Morbidity and Mortality					
Zhang et al., 2018 [41]	Prospective	14,353	Serum Magnesium	<0.70 mmol/L	≥1.0 mmol/L
Fan et al., 2021 [42]	Randomized Control Trial	>10,000	Magnesium Depletion Score	≤2	≥3
Wang et al., 2023 [43]	Cross-Sectional	917 stroke patients	Dietary Magnesium	Quartile ≤ 12 mg/100 kcal/day	Quartile ≥ 18 mg/100 kcal/day
Song et al., 2024 [44]	Cross-Sectional	12,485 Hypertension Patients	Magnesium Depletion Score	≤2	≥3
